# 18q deletion in a cystic fibrosis infant, increased morbidity and challenge for correct treatment choices: a case report

**DOI:** 10.1186/1824-7288-37-22

**Published:** 2011-05-17

**Authors:** Elide Spinelli, Silviana Timpano, Annalisa Fogazzi, Silvia Dester, Susanna Milianti, Rita Padoan

**Affiliations:** 1 Centro Regionale di Supporto per la Fibrosi Cistica, Ospedale dei Bambini, AO Spedali Civili, Brescia, Italy; 2 Clinica Pediatrica, Università degli Studi di Brescia, Brescia Italy; 3 UO di Riabilitazione Specialistica, AO Spedali Civili, Brescia, Italy; 4 UO di Nutrizione clinica e dietetica, AO Spedali Civili, Brescia, Italy; 5 UO Chirurgia Pediatrica, Ospedale dei Bambini, AO Spedali Civili, Brescia, Italy

## Abstract

Cystic Fibrosis (CF) is the most frequent recessive disease of Caucasian patients. Association with other diseases or syndromes has previously been reported. Co-morbidity may be a challenge for clinicians, who have to face more severe problems.

We have described a CF infant, F508del homozygote, diagnosed by neonatal screening, who also had a chromosome 18q terminal deletion [del (18)(q22-qter)]. Some clinical features of the 18q deletion: e.g., cardiopathy, gastro-oesophageal reflux and severe muscular hypotonia, worsened the CF clinical picture and his quality of life, with repeated pulmonary exacerbations and failure to thrive in the first six months of life. The treatment strategy was chosen following an accurate multi-disciplinary team study of overlapping chromosome syndrome and CF symptoms. The use of a gastrostomy device for enteral nutrition together with a new device (Ez-PAP) for chest physiotherapy led to normal growth, a notably reduced hospitalization rate and improved quality of life.

This case shows how co-morbidities worsening the clinical course of a "complicated patient" can be faced thanks to unconventional therapies that represent a challenge for clinicians.

## Background

Cystic fibrosis (CF; MIM #219700) is the most common autosomal recessive disorder among the Caucasian population, there being 1:25 carriers and an incidence of 1:2500-1:4400 [1,2].

CF is caused by mutations in the CFTR gene (7q31.2)(MIM #602421). First cloned in 1989, this encodes a trans-membrane ATP-binding cassette protein functioning as an ion channel, which regulates chloride flow on the apical membrane of epithelial cells [3]. Classical CF clinical symptoms are: recurrent respiratory infections, nasal polyposis, bronchiectasis, exocrine pancreatic insufficiency and male infertility[3].

Several patients have been reported to be affected by CF in association with different diseases or syndromes [4-8]. Clinical features of the genetic syndrome together with CF manifestations create a chimeric disorder requiring a focused therapeutic approach.

Here, we would like to add observations of an additional CF patient with another complicating disease. This is the case of a CF infant with a chromosome 18q deletion (whose incidence is 1/40.000 newborns). No patient has so far been reported to be affected by both 18q- syndrome and Cystic Fibrosis. The estimated probability of finding both syndromes in a single patient is 1/120,000,000. The co-existing pathologies led to a targeted therapeutic choice, which we have presented and discussed.

## Case Presentation

RB, male, aged 2, is the second child born from a couple of Albanian non-consanguineous and healthy parents. He was born after 36 weeks of uncomplicated gestation. The newborn screening was positive for Cystic Fibrosis, with hyper-trypsinogenemia (167.47 ng/ml; cut off 70 ng/ml) and identification of F508del homozygosis.

At birth, several dysmorphisms were noted: i.e., bilateral cryptorchidism, bilateral inguinal hernias, unilateral simian crease, naevus flammeus in fronto-orbital region, congenital cardiopathy (inter-atrial defect), and hypotonia. Standard karyotype performed on peripheral blood lymphocytes and subsequently an *in-situ *fluorescence probe revealed the chromosome 18q22-qter deletion. The parent's karyotype were respectively 46,XX (mother) and 46,XY (father); no cryptic 18q deletion was found by the FISH analysis.

The infant was referred to the Regional Support CF Centre at the Paediatric Department of Brescia (third level hospital) to undergo the follow-up programme for positive screening infant. Sweat test result confirmed CF diagnosis (NaCl value was 108 mEq/l). Both parents were found to be carriers of the F508del mutation, the commonest worldwide mutation (66% of patient alleles). No other family member was affected by the disease.

**Figure 1:**
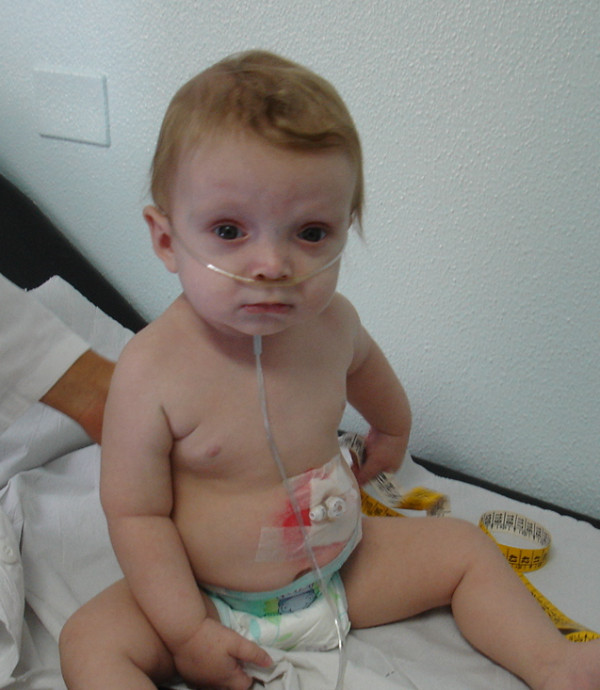
**The patient's usual face**.

Our CF clinic took charge of the child. In addition to the previously reported clinical features, the patient had the following characteristics (Figure 1): wide spaced front, sparse thin hair, deep set eyes, strabismus, mid-face hypoplasia, long philtrum, thin upper lip, retrognatia, dysmorphic low-set ears, tapered digits, proximal thumb, overlapping second and third toe, persistent hypotonia, EEG anomalies and psychomotor delay. Unilateral mammary cystic neo-formations were also detected.

**Figure 2:**
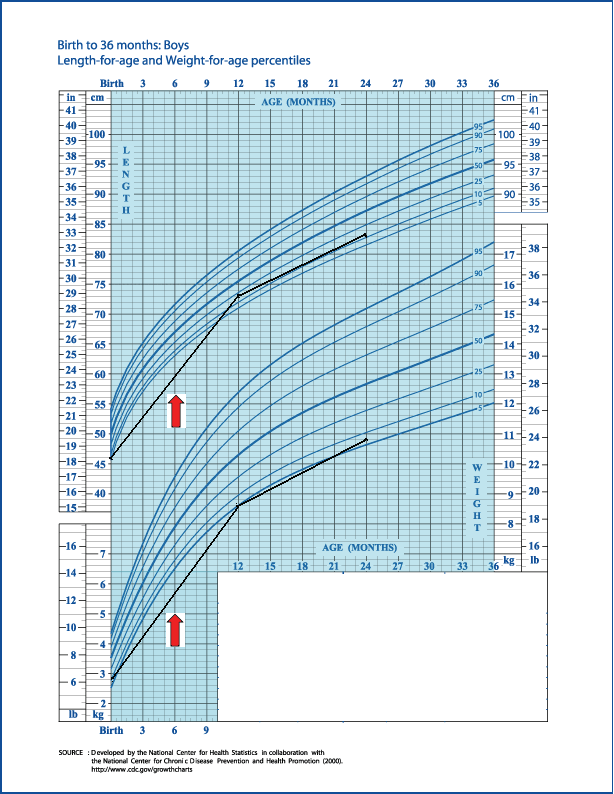
**The patient's growth chart**.

The pancreatic status was insufficient (faecal elastase values < 15 μg/g; severe steatorrhea, assessed by steatocrit > 30%) and, growth was under the 10^th ^percentile for length and < 3^rd ^for weight (Figure 2). Weight/Length (W/L) ratio was < 3^rd ^centile (data not shown).

The cardiopathy the child presents is an inter-atrial defect, ostium secundum type, with left-right shunt and pulmonary hypertension. A marked and diffuse hypotonia, with a severe delay in psychoneural development were also evident.

The standard CF management was started, with pancreatic enzyme supplementation, daily chest physiotherapy by means of PEP-mask, antibiotic therapy when needed as well as salt and vitamin supplementation. For the cardiopathy he received furosemide and continuous oxygen supplementation, moreover Palivizumab was administered monthly from months 2 to 6 [9].

In the first six months of life, he was often hospitalized (once a month) for wheezing, bronchiolitis, respiratory insufficiency (without hypercapnia) with respiratory rate over 100/min requiring CPAP support, recurrent bronchopneumonia, chronic cough. Bronchial secretions microbiology revealed at different times the presence of opportunistic agents as: *Serratia Marcescens, Klebsiella Pneumoniae, Acinetobacter baumannii. Staphilococcus Aureus *and *Pseudomonas Aeruginosa *(PA) responsible for exacerbations. These were treated with intravenous antibiotics and continuous aerosol therapy with tobramicin since the isolation of PA [10].

To avoid strangulation, cryptorchidism and inguinal hernias were surgically treated at three months and during surgery bilateral agenesis of *vasa deferentes *was noted.

Also failure to thrive was evident (W/L was still deeply < 3^rd ^centile). Although low weight and short length might be due both to CF and syndrome, the most evident reason for them was that the child could not ever reach an adequate daily caloric intake for the easy fatigue in suction (due to the hypotonia and high heart rate) and the recurrence of respiratory infections leading to accessional cough and repeated vomiting.

When the patient was 6 months old more aggressive airway clearance management and nutritional programme were decided.

Respiratory function was considerably affected by the severe hypotonia and malnutrition, and the device (PEP-mask) chosen for chest physiotherapy revealed its uselessness with any clearing of bronchial secretions.

We introduced the use of the new device Ez-PAP (Easy Positive Airway Pressure), which while maintaining a positive pressure during the all breathing cycle by high air or oxygen flows, may improve secretion clearance [11]. The Ez-PAP device was used for 3 minutes four times a day, followed by assisted cough. Its use started during hospitalization, when safety and tolerability were careful checked, and then home prescribed, thank to the home oxygen flow availability. Its use did not cause any fatigue for the baby, who complied well to the device, showing improving respiratory pattern and ameliorated chest x-ray images.

Percutaneous endoscopic gastrostomy (PEG) was chosen to provide adequate caloric intake (140 cal/kg/day), given in five daily meals plus nocturnal supplementation with a hydrolyzed diet and MCT. In the same time gastro-oesophageal reflux disease (GORD) was diagnosed and its treatment started and is still ongoing. Pancreatic enzymes were given before each meal and at start and in the middle of nocturnal supplementation, at doses able to correct steatorrhea.

In Figure 2 the catch up curves of weight and length are shown (arrows indicate when enteral nutrition started and final length and weight at 24 months) leading to the 3-10^th ^centile for weight and on the 10-25^th ^centile for length at 18 and 24 months (W/L 10-25^th ^centile).

After the Ez-PAP device was introduced and gastrostomy (GS) allowed the adequate caloric intake, a significant improvement in clinical condition was evident, with only one more hospitalization till two years.

**Figure 3:**
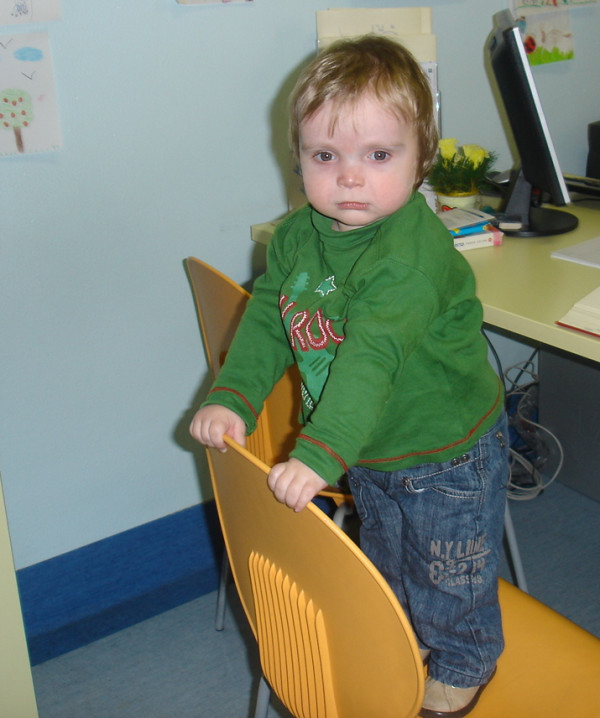
**The patient at 18 months**.

Daily neuromuscular physiotherapy was also performed. The child was able to sit up unaided at 12 months, started assisted stand-up position at 18 months (Figure 3) and assisted walking at 24 months.

At 24 months a swallowing study was performed with normal results. Oral feeding was encouraged while maintaining enteral supplementation by GS to reach adequate caloric intake. Speech therapy was started to improve swallowing and speech. Oxygen therapy was no longer needed.

A syndrome based follow-up was also started. Auxo-endocrinologic, oculist (showing hypermetropia), ENT, audiologist, orthopaedic, cardiologic (proving reduction of pulmonary hypertension), immunologic (IgA deficiency was detected), and neuropsychiatric evaluations (assessing the cognitive delay) evaluations, were all regularly scheduled. Cerebral MRI has not yet been performed for its high anaesthesiologic risk.

## Discussion

This case report underlies how two co-existing diseases usually complicate each other's clinical symptoms. Cystic Fibrosis and 18q distal deletion syndrome were found together in a patient and their clinical consequences have been described for the first time in this case report.

The 18q- syndrome is determined by the terminal deletion of the chromosome 18q arm. The length of the deletion can vary from patient to patient, the commonest involved bands being 18q21-qter. First described in 1964 by DeGrouchy *et al.*, [12] the syndrome attributes several distinctive features: short stature, hypotonia, mental retardation, delayed developmental milestones, mid-face hypoplasia, "carp-like" mouth, palatal abnormalities, tapered digits, proximal thumbs, hearing impairment, genitourinary malformations (cryptorchidism, *labra maiora *absent) and umbilical and inguinal hernia [MIM #601808]. Immunoglobulin A and M deficiencies [13,14] have also been described. Ocular anomalies such as strabismus, anterior optic segment malformations, nystagmus and myopia have also been reported in several patients [15,16]. A variety of neurological malformations and disorders are also part of the syndrome: myelination disturbances, absent *corpus callosus*, holoprosencephaly, seizures and EEG pattern anomalies and psychiatric disorders (Rett syndrome, autism, depression, anxiety, mania, psychosis) [17-20].

As regard the correct diagnosis, our patient's case history underlines the importance of neonatal screening, that may allow early detection of CF in infants whose co-morbidities would otherwise dramatically delay the diagnosis. Up to now, however, it is not performed by all Italian Regions, and in 2009, 22% of newborn Italian population did not undergo a CF neonatal screening (report of the Italian Society for the Study of Inherited Metabolic Diseases and Newborn Screening, 2010). Thus in Regions where it is not performed, in all infants with poor growth, and/or recurrent respiratory infections, it is mandatory to exclude the CF disease.

Our patient's CF depending life prognosis and his quality of life was worsened by syndrome symptoms. Mental retardation was another complicating feature: self-care and daily home therapy will require continuous domestic and hospital surveillance.

Muscular hypotonia made the application of usual chest physiotherapy device to our patient impossible. Recently, in order to improve mucociliary clearance and lung expansion, a new device called EzPAP has been proposed [11]. It produces a positive airway pressure throughout the breathing cycle via flow from an air or oxygen flow meter and flow amplification. It is used for atelectasis prevention and treatment and up to now the major experience is on the treatment of postsurgery atelectasis in adults [21]. The use of this device in our patient prevented subsequent respiratory infections without fatiguing the patient, given that no respiratory muscle force is required. No information is currently available on Ez-PAP use in children or CF patients. A preliminary experience shows the device might be a solution for removing bronchial secretions in selected patients [22].

GS for enteral nutrition is commonly used for CF and patients suffering from neuromuscular disorders and disability [23] as well as CF patients needing adequate nutritional support. Our patient presented all the clinical criteria for starting a chronic enteral nutrition supplementation, which showed its usefulness in allowing a normal growth pattern.

## Conclusion

Several factors together have contribute to improve our patient's clinical condition: a better nutritional status, the continuous use of antibiotics, therapy of GORD and the use of the new device, all reduced the recurrence of respiratory exacerbations and the need of hospitalizations.

Our patient's history stresses the importance of being ready to face clinical conditions which can be severely complicated by co-existing diseases or genetic syndromes and how this goal can be achieved through a multi-disciplinary team of experts.

It is imperative to understand all therapeutic choices available for CF patients and the need to share all treatment chosen for CF patients with associated severe disease. These "brave" choices can improve treatment for patients with CF alone. The same practice could be helpful when facing to clinical conditions different from CF, but leading to similar conditions, as malnutrition and recurrent respiratory infections by bacteria as *Pseudomonas aeruginosa*, that are commonly seen in neuropsychiatric clinics: the use of gastrostomy, chest therapy and standard CF antibiotic protocols against opportunistic agents may be used with positive outcome and prognosis improvement also in these patients.

Finally, the work of a multidisciplinary cystic fibrosis therapeutic team is mandatory to improve the treatment of such severe and complicated clinical cases.

## Consent

Written informed consent was obtained from the patient's relatives for publication of this case report and the patient's images.

## Competing interests

The authors declare that they have no competing interests.

## Authors' contributions

ES, medical geneticist stated the syndrome's follow up, wrote and discussed the manuscript; ST, paediatrician, participated in clinical management during hospitalizations; AF, chest therapist, treated the patient; SD, dietician, stated dietetic treatment; SM, surgeon, performed surgical interventions; RP, paediatrician, has the responsibility of the patient clinical management and participated in case discussion and writing the manuscript.

All authors read and approved the final manuscript.
